# Editorial: The physical environment and health: implications for the planning and management of healthy cities

**DOI:** 10.3389/fpubh.2023.1245561

**Published:** 2023-08-01

**Authors:** Linchuan Yang, Baojie He, Long Cheng, Ruoyu Wang, Yibin Ao

**Affiliations:** ^1^Department of Urban and Rural Planning, School of Architecture, Southwest Jiaotong University, Chengdu, China; ^2^Centre for Climate-Resilient and Low-Carbon Cities, School of Architecture and Urban Planning, Chongqing University, Chongqing, China; ^3^School of Transportation, Southeast University, Nanjing, China; ^4^UKCRC Centre of Excellence for Public Health, Centre for Public Health, Queen's University Belfast, Belfast, United Kingdom; ^5^College of Environment and Civil Engineering, Chengdu University of Technology, Chengdu, China

**Keywords:** built environment, living environment, human health, physical health, mental health, healthy city, wellbeing

In recent years, there has been a significant upsurge in the demand for a high quality of life, as individuals strive to lead healthy and fulfilling lives. A multitude of health behaviors, outcomes, and issues (e.g., physical activity, obesity, diabetes mellitus, stress, depression, high blood pressure, and health inequalities and inequities) have emerged as major concerns, capturing the attention of governments, societies, academia, and other stakeholders (1–5). Recognizing the pivotal role of the physical environment—the geographic area and surrounding factors—in shaping human health behaviors and outcomes, extensive research has focused on understanding the complex connections between the two.

The physical environment encompasses various elements that directly or indirectly influence our health and overall wellbeing. Many aspects of the physical environment, such as air and water quality, noise pollution, exposure to hazardous substances, housing conditions, indoor environments, and access to open/green spaces and recreational areas, contribute to human health. Therefore, gaining a comprehensive understanding of how these aspects interact with health behaviors and outcomes is of utmost importance (6–10).

The concept of a “healthy city” was introduced by the World Health Organization in the last century and has since been widely discussed, explored, and advocated worldwide. The notion of a healthy city emphasizes the importance of improving the physical environment as a critical element in achieving optimal health and wellbeing for urban populations. The physical environment plays a crucial role in shaping health behaviors and outcomes within urban settings, making it essential to prioritize its enhancement as part of broader healthy city initiatives. Scientific advancements are constantly anticipated to foster collaboration between governments and society toward the establishment of healthy cities.

Furthermore, the recent outbreak of the coronavirus disease 2019 (COVID-19) has had an unprecedented impact on societies worldwide (11, 12). The rapid spread of the virus has underscored the need for cities to develop strategies that integrate the fight against COVID-19 with the framework of healthy cities (13–15). It is crucial to gather and summarize the experiences of cities in their response to the pandemic, encompassing not only strategic frameworks but also practical implementation. This includes a specific focus on enhancing the urban physical environment to mitigate the transmission of the virus, promote public health interventions, and ensure the resilience of cities in the face of future health crises.

To address these pressing concerns by gathering research and insights from diverse perspectives and to catalyze in-depth and thoughtful discussions on the intricate relationship between the physical environment and human health, this Research Topic was initiated in July 2022. (In *Frontiers* journals, a “Research Topic” corresponds to what is commonly referred to as a “special issue” or “virtual special issue” in many other journals.) It is supported by four *Frontiers* journals: *Frontiers in Public Health, Frontiers in Ecology and Evolution, Frontiers in Environmental Science*, and *Frontiers in Sociology*. It serves as a platform for disseminating the latest insights and discoveries in the realm of the physical environment and human health, particularly those with profound theoretical, methodological, and practical implications. The primary objectives are to promote scientific progress, offer research support for policy development and evaluation, and stimulate immediate attention from governments, businesses, researchers, and individuals.

After undergoing a rigorous review process, a total of 67 papers have been selected for inclusion in this Research Topic. Among these papers, 63 are classified as “Original Research,” showcasing the innovative contributions made by the authors. One paper falls under the “Methods” category, emphasizing the importance of developing robust methodologies for studying the intricate relationship between the physical environment and human health. Additionally, three papers are designated as “Review” articles, synthesizing existing knowledge in the field and providing valuable insights. Moreover, the collected papers have been published across the four supporting journals, with 58 appearing in *Frontiers in Public Health*, 4 in *Frontiers in Ecology and Evolution*, 4 in *Frontiers in Environmental Science*, and 1 in *Frontiers in Sociology*. This diverse distribution of the 67 papers across these journals reflects the interdisciplinary nature of the topic, as scholars from diverse disciplines have brought forth their expertise and perspectives.

The selected papers are authored by over 300 scholars. The authors are from a wide range of countries, emphasizing the global scope of research in the field of the physical environment and health. Scholars from many countries such as China, the United States, the United Kingdom, Canada, Finland, Switzerland, Australia, Japan, India, and Nepal have contributed to this special issue. The inclusion of researchers from diverse cultural backgrounds enhances the richness and breadth of perspectives presented in the papers.

The collected papers cover a wide array of topics, addressing crucial issues related to the physical environment and multi-dimensional human health (e.g., physical and mental health). The topics include but are not limited to COVID-19 (Dubey et al.; Tang et al.), the physical environment and health behaviors (e.g., Yang et al.; Zeng et al.), the physical environment and health outcomes (e.g., Zhang et al.; Zhu et al.), urban public spaces (e.g., Chen et al.; Wei et al.), and evacuation risks (e.g., Li et al.; Lv et al.). To sum up, the diverse range of topics covered in this collection of papers reflects the interdisciplinary nature of research in the physical environment and human health. Scholars from fields such as public health, urban planning and design, architecture, landscape architecture, human geography, public administration, environmental science, and urban management have contributed to this special issue, highlighting the collaborative effort required to address the complex challenges at the intersection of the physical environment and human health.

Papers included in this Research Topic and a sea of previous studies highlight the critical importance of addressing physical environmental factors that can significantly impact human health and overall wellbeing. The findings underscore the urgent need to prioritize the protection of public health by creating a health-supportive environment. This necessitates the implementation of policies and regulations that enhance air and water quality, advocate for sustainable and healthy housing options, mitigate exposure to hazardous substances, manage noise pollution, and preserve and create green spaces. By taking proactive measures in these areas, we can positively influence the health outcomes of individuals and communities.

To further advance our understanding and approach in this field, numerous research directions can be envisioned for future exploration. These include (1) investigating the connections between the physical environment (e.g., the built environment, extreme heat/cold, urban flooding, water/air/soil pollution, the quality of transport/food/living/housing) and health behaviors/outcomes, (2) inequalities and inequities that exist within these connections (e.g., how different populations, particularly marginalized communities, are disproportionately affected by environmental factors and how these disparities can be addressed to promote health equity?), (3) health implications of new city concepts like healthy cities and 15-min cities, (4) health-oriented urban physical examination and design, (5) COVID-19 implications for the planning and management of healthy cities, (6) urban resilience in the face of pandemic disruptions and climate change, and (7) resilience management, governance, and policy formation for healthy cities. Finally, the translation of research findings into practical applications remains a persistent challenge. Bridging the gap between research and practice is essential to ensure that evidence-based interventions and policies are effectively implemented. Further research should focus on identifying strategies to enhance the dissemination and application of research findings, fostering collaborations between researchers, policymakers, and practitioners, and fostering knowledge exchange platforms. We can hope that after gaining valuable insights into designing health-supportive urban environments, evidence-based interventions can be effectively implemented for the benefit of individuals and communities.

## Author contributions

LY: conceptualization, funding acquisition, writing-original draft, and writing-review and editing. BH, LC, RW, and YA: writing-review and editing. All authors listed have made a substantial, direct, and intellectual contribution to the work.
